# Creation and Pilot-testing of Virtual Patients for Learning Oncologic Emergency Management

**DOI:** 10.7759/cureus.6206

**Published:** 2019-11-20

**Authors:** Ziad Simon Fawaz, Nancy Posel, Benjamin T Royal-Preyra, Julia Khriguian, Joanne Alfieri

**Affiliations:** 1 Radiation Oncology, McGill University Health Center, Montreal, CAN; 2 Medical Education, McGill University Health Centre, Montreal, CAN; 3 Radiation Oncology, McGill University Health Centre, Montreal, CAN

**Keywords:** oncology, emergency, simulation, education, module, virtual

## Abstract

Purpose or objective

Management of oncologic emergencies becomes critical at the start of the second year of a radiation oncology residency. Considering the limited exposure to oncology in the medical school curriculum, this knowledge gap needs to be filled prior to managing real patients. The aim of this project was to create virtual patients (VPs) to ease this transition and improve learner readiness for independently managing oncologic emergencies on call.

Material and methods

A curriculum mapping exercise was done to identify gaps. The main oncologic emergencies that needed to be addressed were selected for development of the modules. Review of the key concepts for management was elucidated and validated. These included history, physical examination, imaging interpretation, staging, as well as anatomy, epidemiology, pertinent literature, differential diagnosis, prognostication, radiation treatment planning, summarizing, and patient- and peer-communication skills. Clinical vignettes were then designed, in collaboration with a virtual patient education expert, to mimic the clinical presentation and evolution of a typical patient for three common oncologic emergencies: spinal cord compression, superior vena cava syndrome, and tumor-induced hemorrhage.

Results

Three virtual modules were developed: spinal cord compression, superior vena cava syndrome, and tumor-induced hemorrhage. Each case included 25 to 30 vignettes that participants progressed through, with a total estimated completion time of 30 to 45 minutes. Each node branched out to provide a detailed answer and explanation of the key concept. Figures were included to mimic real patients and to provide a more authentic learning experience. The modules also included quantitative pre- and post-testing assessments, including multiple-choice questions, true or false, fill in the blank, short answers, and text response. The cases were then transcribed onto a virtual patient simulation platform. Following completion of the module, a report was generated for each individual learner to track all responses and used as the assessment tool. The pilot test showed an increase of 28% in the pre-to-post-test results in a cohort of nine residents. The mean pre-test result of 58% increased to a mean post-test result of 86% (range: 70-100%) after completing the three modules.

Conclusion

VPs can be used for learning the management of oncologic emergencies and can be done on a simulation-based learning platform. The modules can be used as both, a learning and an assessment tool for junior residents. The results of the pilot test show a significant improvement in knowledge acquisition between pre- and post-test scores after completion of the three modules.

## Introduction

Exposure to radiation oncology ranges from non-existent to very limited in medical school curriculums [1-21]. Typically, medical students progress from the first part of their training by covering the basics such as physiology and pharmacology, which is then followed by clinical exposure in a hospital or clinical setting [3-6].

Most subspecialties are well covered in the medical curriculum. However, radiation oncology is either never introduced in a student’s training preceding residency, or introduced briefly as part of their internal medicine core rotation or oncology elective [9-10]. A review by Ben Mustapha showed that the median number of hours devoted to radiation oncology teaching in undergraduate medical education was only 10 hours and ranged from 2 to 60 hours [4, 18]. When the teaching did occur, it was not uniform, limited in time, and considered an underrepresented component of undergraduate medical education [2, 8, 13].

This situation, of limited exposure to the specialty, leaves junior radiation oncology residents ill-prepared for being on call and managing oncologic emergencies early on in their radiation oncology training.

Junior residents start their postgraduate medical training with a year of fundamental clinical training including medical and surgical subspecialties, radiology, pathology, and palliative care [7, 11, 20]. These core rotations are fundamental in building the knowledge required to become a competent radiation oncologist who must be comfortable diagnosing common medical and surgical conditions, interpreting imaging, and managing complex patients. Nevertheless, exposure to the management of oncologic emergencies during this period is lacking.

To address this gap and supplement current training, the goal of this project was to develop a learning tool to ensure adequate medical competence of junior radiation oncology residents to ensure safety for patients presenting with oncologic emergencies. This project encompasses the creation and pilot-testing of virtual patients’ modules.

## Materials and methods

A curriculum mapping exercise was done in order to identify knowledge gaps. The main oncologic emergencies that needed to be addressed were selected for development of learning modules. Review of key concepts for management was elucidated and validated. Clinical vignettes were then designed, with collaboration of a virtual patient education expert, to mimic the clinical presentation and evolution of a typical patient exhibiting signs and symptoms of three common oncologic emergencies: spinal cord compression, superior vena cava syndrome and bleeding secondary to a malignancy. Each virtual patient case was divided in four sections: research consent, pre-test, virtual patient, and post-test. The project was completed with the support of the Center for Medical Education.

The first section of each case consisted of a research informed consent statement. This statement specified that while completion of the modules was obligatory for the residency training program, participation in the research portion was voluntary. It also included a statement on the confidentiality of all the information gathered.

The second section consisted of a quantitative pre-test. The answers were not provided at this stage; however, they were made available once each module was completed. Each pre-test included either eight or 10 questions with multiple choice, true or false, short-answer, text response or fill-in-the-blank questions.

The third section consisted of the virtual patients (VPs). Each VP is presented, and an initial question regarding the next most important action is posed. The learner then progresses through the VP case with questions and answers on each of the key elements of an assessment as described in Table 1. The learner does not progress through the case until the correct answer is chosen. A correct answer is provided, including a detailed description and explanation of the learning concept, as well as a supporting reference. Figures and tables are embedded in the answers. Images are included to mimic real patients and to provide a more authentic learning experience.

**Table 1:** Learning concepts included in the modules

History
Physical examination
Imaging interpretation
Staging
Anatomy
Epidemiology
Pertinent literature
Differential diagnosis
Prognostication
Radiation planning
Summarizing
Communication skills

A medical illustrator created original images to support the VP narrative and help reinforce each of the elements being taught. Once the main elements are covered, the learner is asked to summarize the case in writing, as if presenting to an attending physician. This step is followed by a description of the radiation management plan, including the main elements of a radiation prescription as seen in Table 2. The VPs end with an open-ended question where learners are asked to assess their own performance and identify take-home messages.

**Table 2:** Component of a radiation treatment prescription

Diagnosis
Intent
Treatment site
Dose and fractionation
Simulation
Number of plans
Fusion
Technique
Imaging limits
Immobilization
Position
Image guidance
Field positioning
Volumes

The fourth section consists of a post-test which repeats the same questions from the pre-test. The answers from the pre- and post-test were used for data analysis. The number of participants and the time to module completion for each participant were recorded. The mean scores of the pre- and post-tests were collected, and means were calculated and compared.

## Results

Creation of the modules

The three scripts were written to include the main concepts that are fundamental to the management of the case. The scripts were subsequently transcribed on DecisionSim Kynectiv, a simulation-based learning platform used for assessment, education, and training, and is accessible to the learner through a personal account. The VPs were created, and their transcription was followed by iterative corrections by experienced radiation oncologists.

**Figure 1:**
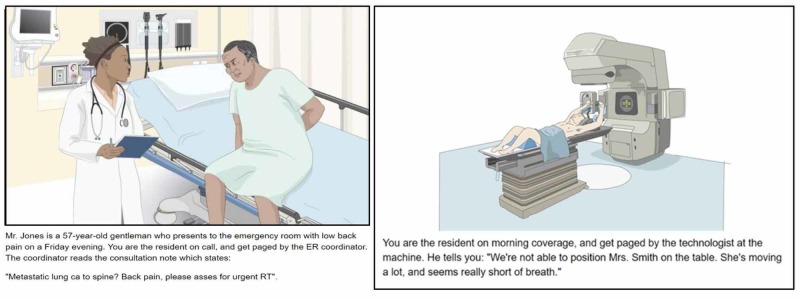
Initial case presentation

Each VP consisted of 25 to 30 screens to progress through, with a total estimated completion time of 30 to 45 minutes. The VP started with an initial case presentation and an image as seen in Figure 1.

**Figure 2:**
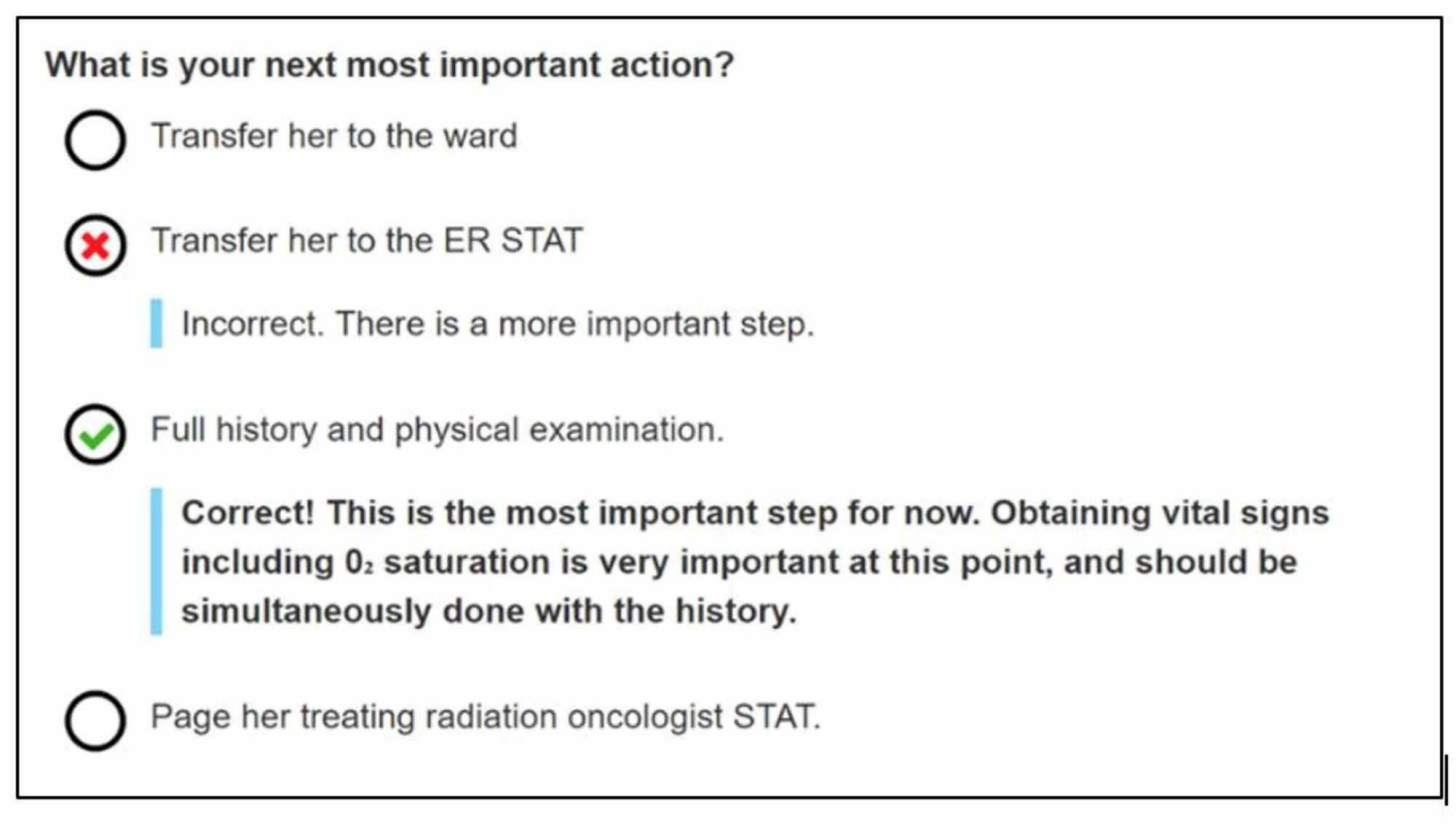
Example of a management question

The year of training, the start time, the end time, the pre-test result, and the post-test result of each learner are recorded. A navigation bar in the module is also available so that the learner could monitor their progression. Correct responses are immediately fed back to the learners as they progress through each VP as shown in Figure 2.

The learner is not able to progress through the VPs until the correct response is given. However, the number of attempts is not limited. Completion of the module is recorded, and a report is generated for each individual learner tracking all responses which allows for it to be used as an assessment tool. Qualitative survey data, regarding user-friendliness and learner satisfaction, was also collected.

Pilot-testing of the modules

The pilot test consisted of nine radiation oncology trainees out of 10 trainees who consented to participate in the research component of this project. An introductory 30-minute teaching session on oncologic emergencies was given by a radiation oncologist and was followed by the three VP cases. Forty-five minutes were allotted for each virtual patient. The total teaching session, including the introduction and the three cases, lasted two and a half hours.

The mean time to VP completion was 32 minutes with a mean of 36 minutes for the spinal cord compression VP, 31 minutes for the superior vena cava syndrome VP, and 28 minutes for the bleeding VP.

The pre-test was initiated after the introductory teaching session. The mean pre-test result for all VPs was 58% (range 50-70%) and increased to a mean of 86% (range 70-100%) in the post-test after completion of the three modules. All the learners improved their test score on the post-test.

**Figure 3:**
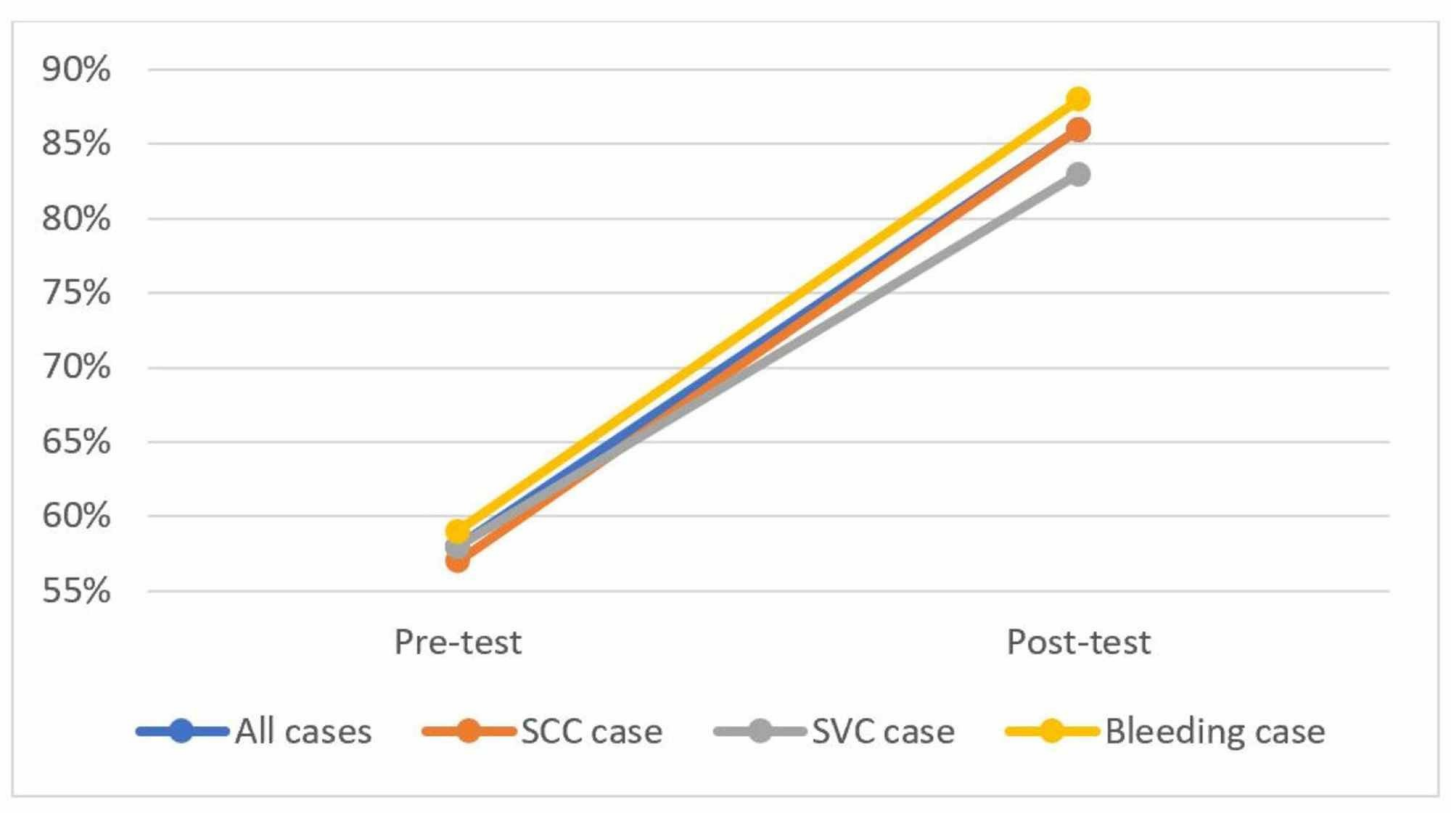
Mean pre- and post-tests results based on the case

There was little variability in the pre-test results between each of the three cases with a mean pre-test result of 57% for the spinal cord compression VP, 58% for the superior vena cava syndrome VP, and 59% for the bleeding VP. The same observation was made with the post-test results with a mean post-test result of 86% for the spinal cord compression VP, 83% for the superior vena cava syndrome VP, and 88% for the bleeding VP (Figure 3).

**Figure 4:**
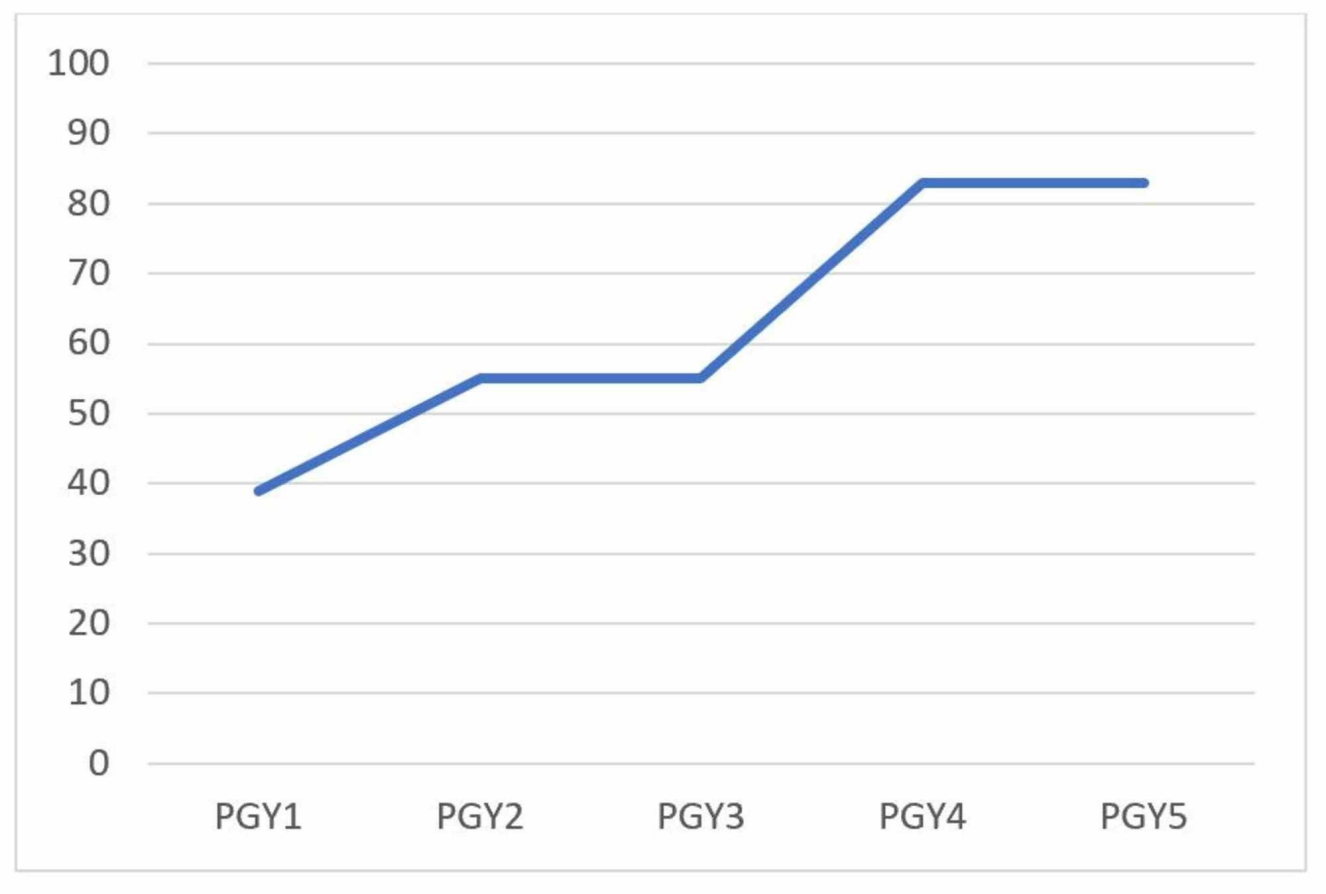
Pre-test results based on level of training

Plotting the mean pre-test score by level of training shows a trend for higher scores with increasing seniority as seen in Figure 4 with less increase in scores after completing of the modules. The satisfaction was rated as high by the learners. The comments in the feedback section were favorable about the assessment component, the dedicated teaching time for completion, the thorough explanations of the concepts, and the user-friendliness. For example, one learner stated, “the modules were very easy to follow, and provided a thorough review of the topic”.

## Discussion

This project shows that the creation and implementation of VPs to teach oncologic emergencies in a radiation oncology training program is feasible and yields positive results. It is a practical and relatively easy way to transfer domain knowledge and assess its virtual application. In addition, once created, these cases can be used as needed without any further time allocation. They can also easily be modified if there are practice changes. Our pilot test showed a significant improvement in the understanding of the management of spinal cord compression, superior vena cava syndrome and tumor hemorrhage. This implies that VPs can provide an additional learning strategy to support post-graduate residents.

Evaluation

The virtual simulation platform chosen was revealed to be a convenient and reproducible tool. The use of such tools has significantly increased with the integration of e-learning in medical education and the use of clinical problem-solving software, which has now more than doubled in the past decade [14, 21]. The effectiveness of simulation-based medical education was compared to traditional clinical education, and was found to be more effective in a comparative meta-analysis by McGaghie et al. in 14 studies published between 1990 and 2010 [16].

Methods to assess the effectiveness of the VPs were also considered in their development. Noesgaard and Ørngreen published on the use of e-learning with a review on the definitions, methodologies and factors that promote its effectiveness. There were 92 papers reviewed that revealed 19 distinct ways of defining effectiveness with the most common being the overall “learning outcome”. They showed that 73% of the studies used quantitative methods to assess learning with the most effective being pre- and post-tests. The formulation of the assessment included multiple choice questions, true or false, drag-and-drop, fill-in-the-blank and matching. Factors that were found to influence effectiveness were that the teaching method was applicable to practice, measured the cognitive load, included problem-based learning, provided control to the learner, and included elements of collaboration and communication [18, 19]. These factors were incorporated into the design of the VPs.

Our results highlight the effectiveness of the use of VPs as a new and innovative strategy for teaching as part of blended learning. This blended learning model can include virtual tools, webinars, and face-to-face teaching. It allows for a cohesive learner experience and has been increasing in the past decade due to technological advances and the need for flexibility in learning [17]. This model can also be integrated in the Kirkpatrick Four-level model of evaluation which includes the reaction of the learner, learning the concepts, behavioral application of the knowledge, and results in terms of performance [3]. The first two levels are demonstrated by this study, and further evaluation would be required to demonstrate levels three and four.

Assessment

From the literature, it was ascertained that the VPs needed to contain a few crucial elements. A systematic review by Issenberg et al. discussed an educational intervention or assessment of a high-fidelity simulation by analyzing learner outcomes quantitatively [12]. Factors that lead to effective learning included a simulation integrated into the set curriculum and executed in a controlled environment, as well as feedback being provided during the learning experience. These three elements were incorporated in our virtual mid-fidelity patients.

Kleinert et al. also reported that web-based virtual patient simulation improves clinical reasoning by increasing competence and raising learners' motivation [15]. Berman et al. have added on the importance of using virtual patients to promote deep learning, capture intrinsic motivation, and promote retention [5]. This form of teaching was also found to be complementary to competency-based education which is being implemented in multiple training programs [7]. The qualitative data we collected suggested that our learners enjoyed the experience which confirms the author findings with feedback comments such as "helped me realize that I have to review this topic", "effective method to learn this management", and "great overview of the topic".

Limitations

Although the results of this project are promising, they cannot achieve significance due to the small cohort size of nine learners. This limited cohort number limits the statistical power of the study and highlights the need to replicate this study in the next academic year. Another limitation is the absence of a delayed post-test at three months to assess knowledge retention, recall, and deep learning.

Our approach using VPs provides a closer representation of real-life situations and can have a significant impact on theoretical and procedural knowledge, which could also lead to improved patient outcomes. Although patient outcomes could not be measured and were outside the scope of this project, further studies on this topic could demonstrate higher levels of the Kirkpatrick framework, and provide important insight on the most effective learning methods that impact patient care.

## Conclusions

A blended learning approach was successful in supporting the learning of management of oncologic emergencies. VPs are an effective tool to apply this approach in a residency program and can be done on a simulation-based learning platform. This method can be used both as an assessment, as well as a teaching tool for junior residents using a blended approach of online learning and face-to-face teaching. The results of the pilot test show that each VP can be completed in a single half-hour teaching session, and can lead to a significant improvement in the knowledge and performance of junior residents.

## References

[1] Agarwal A, DeNunzio NJ, Ahuja D, Hirsch AE (2014). Beyond the standard curriculum: a review of available opportunities for medical students to prepare for a career in radiation oncology. Int J Radiat Oncol Biol Phys.

[2] Arenas M, Sabater S, Biete A, Lara P, Calvo F (2018). Radiation oncology teaching programmes as part of the undergraduate degree in medicine in Spanish universities: the need for an update of the contents and structure. J Cancer Educ.

[3] Bates R (2004). A critical analysis of evaluation practice: the Kirkpatrick model and the principle of beneficence. Eval Program Plann.

[4] Ben Mustapha S, Meijnders P, Jansen N, Lakosi F, Coucke P (2019). The status of radiation oncology (RO) teaching to medical students in Europe. Clin Transl Radiat Oncol.

[5] Berman N, Durning S, Fischer M, Huwendiek S, Triola M (2016). The role for virtual patients in the future of medical education. Acad Med.

[6] Branch WT Jr, Paranjape P (2002). Feedback and reflection: teaching methods for clinical settings. Acad Med.

[7] Royal College of Physicians and Surgeons of Canada (2017). Competence by Design: What You Need to Know. A Resident’s Guide. http://www.royalcollege.ca/rcsite/documents/cbd/cbd-residents-guide-e.pdf.

[8] Royal College of Physicians and Surgeons of Canada (2014). Specialty Training Requirements in Radiation Oncology. http://www.royalcollege.ca/rcsite/documents/ibd/radiation_oncology_str_e.pdf.

[9] Golden DW, Spektor A, Rudra S (2014). Radiation oncology medical student clerkship: implementation and evaluation of a bi-institutional pilot curriculum. Int J Radiat Oncol Biol Phys.

[10] Hirsch AE, Handal R, Daniels J (2012). Quantitatively and qualitatively augmenting medical student knowledge of oncology and radiation oncology: an update on the impact of the oncology education initiative. J Am Coll Radiol.

[11] Hirsch AE, Singh D, Ozonoff AI, Slanetz PJ (2007). Educating medical students about radiation oncology: initial results of the oncology education initiative. J Am Coll Radiol.

[12] Issenberg S, Mcgaghie WC, Petrusa ER, Gordon DL, Scalese RJ (2005). Features and uses of high-fidelity medical simulations that lead to effective learning: a BEME systematic review. Med Teach.

[13] Jagadeesan VS, Raleigh DR, Koshy M, Howard AR, Chmura SJ, Golden DW (2014). A national radiation oncology medical student clerkship survey: didactic curricular components increase confidence in clinical competency. Int J Radiat Oncol Biol Phys.

[14] Kim S (2006). The future of e-learning in medical education: current trend and future opportunity. J Educ Eval Health Prof.

[15] Kleinert R, Heiermann N, Plum PS (2015). Web-based immersive virtual patient simulators: positive effect on clinical reasoning in medical education. J Med Internet Res.

[16] McGaghie WC, Issenberg B, Cohen ER, Barsuk JH, Wayne DB (2011). Does simulation-based medical education with deliberate practice yield better results than traditional clinical education? A meta-analytic comparative review of the evidence. Acad Med.

[17] Mirriahi N, Alonzo D, Fox B (2015). A blended learning framework for curriculum design and professional development. Res Learn Technol.

[18] Noesgaard SS, Ørngreen R (2015). The effectiveness of e-learning: an explorative and integrative review of the definitions, methodologies and factors that promote e-learning effectiveness. EJEL.

[19] Saeed S (2018). Undergraduate medical education and radiation oncology: current concerns and effective initiatives. Appl Rad Oncol.

[20] Vaughn L, Baker R (2001). Teaching in the medical setting: balancing teaching styles, learning styles and teaching methods. Med Teach.

[21] Willis RE, Sickle KRV (2015). Current status of simulation-based training in graduate medical education. Surg Clin North Am.

